# PMA-Induced THP-1 Macrophage Differentiation is Not Impaired by Citrate-Coated Platinum Nanoparticles

**DOI:** 10.3390/nano7100332

**Published:** 2017-10-17

**Authors:** Francesca Gatto, Roberta Cagliani, Tiziano Catelani, Daniela Guarnieri, Mauro Moglianetti, Pier Paolo Pompa, Giuseppe Bardi

**Affiliations:** 1 Nanobiointeractions & Nanodiagnostics, Istituto Italiano di Tecnologia, Via Morego 30, 16163 Genova, Italy; francesca.gatto@iit.it (F.G.); roberta.cagliani@iit.it (R.C.); daniela.guarnieri@iit.it (D.G.); Pierpaolo.Pompa@iit.it (P.P.P.); 2 Department of Engineering for Innovation, University of Salento, Via per Monteroni, 73010 Lecce, Italy; 3 Department of Chemistry and Industrial Chemistry, University of Genova, Via Dodecaneso 31, 16146 Genova, Italy; 4 Electron Microscopy Laboratory, Istituto Italiano di Tecnologia, Via Morego 30, 16163 Genova, Italy; tiziano.catelani@iit.it; 5 Nanobiointeractions & Nanodiagnostics, Center for Bio-Molecular Nanotechnologies, Istituto Italiano di Tecnologia, Via Barsanti, 73010 Arnesano, Lecce, Italy; mauro.moglianetti@iit.it

**Keywords:** macrophages, platinum nanoparticles, differentiation, cytokines, chemokines

## Abstract

The innate immune system consists of several complex cellular and molecular mechanisms. During inflammatory responses, blood-circulating monocytes are driven to the sites of inflammation, where they differentiate into tissue macrophages. The research of novel nanomaterials applied to biomedical sciences is often limited by their toxicity or dangerous interactions with the immune cell functions. Platinum nanoparticles (PtNPs) have shown efficient antioxidant properties within several cells, but information on their potential harmful role in the monocyte-to-macrophage differentiation process is still unknown. Here, we studied the morphology and the release of cytokines in PMA-differentiated THP-1 pre-treated with 5 nm PtNPs. Although NP endocytosis was evident, we did not find differences in the cellular structure or in the release of inflammatory cytokines and chemokines compared to cells differentiated in PtNP-free medium. However, the administration of PtNPs to previously differentiated THP-1 induced massive phagocytosis of the PtNPs and a slight metabolism decrease at higher doses. Further investigation using undifferentiated and differentiated neutrophil-like HL60 confirmed the harmlessness of PtNPs with non-adherent innate immune cells. Our results demonstrate that citrate-coated PtNPs are not toxic with these immune cell lines, and do not affect the PMA-stimulated THP-1 macrophage differentiation process in vitro.

## 1. Introduction

Immunity is characterized by innate and acquired defense systems that allow protection against pathogenic hazards [1]. Several active molecules and fast-responding leukocytes belong to the innate immune system, such as neutrophils, NK cells and monocyte/macrophage phagocytes. These cells guarantee a prompt and unspecific response, which is triggered by different internal or external biochemical mediators. In particular, monocytes circulating in the bloodstream react to chemo-attractant inflammatory stimuli by migrating to the site of infection, where they differentiate into tissue-specific macrophages able to phagocyte and eliminate the threats. Furthermore, in the case of a viral infection, viral proteins are exposed on the macrophage membrane to prime the lymphocyte-dependent adaptive immune response and amplify it by the release of specific cytokines. The monocyte-to-macrophage differentiation process represents an extremely sensitive step for correctly proceeding with an appropriate immune reaction in all types of infection. Potential impairment of immune mechanisms, like those described earlier, should be always carefully considered before developing novel medical treatments and therapeutic approaches, including the use of nanotechnologies [2,3].

Among the nanomaterials with feasible biomedical applications, platinum nanoparticles (PtNPs) demonstrate efficient antioxidant activity due to their intrinsic catalytic properties [4]. However, metallic NPs have often rose concerns on their potential toxicity, mostly induced by the loss of toxic ions, the presence of synthesis by-products or the use of unsafe coating materials [5]. NP coatings are very important for the potential immunotoxicity of the NPs, since they can directly bind to immune receptors or adsorb active molecules that change the immunological identity of the particles [6,7]. Our group recently synthesized citrate-coated PtNPs demonstrating efficient intracellular radical oxygen species (ROS) scavenging activity and a high degree of cytocompatibility [8]. The safety and antioxidant activity of PtNPs have also been reported in murine RAW 264.7 macrophages in vitro with a significant reduction of ROS production and LPS-induced inflammatory cytokine release [9]. These results suggest a potential use of PtNPs as synthetic enzymes (“nanozymes”) with antioxidant activity based on their efficient catalytic properties. However, deep investigation on PtNP interaction with human immune cells and their biology is still lacking.

In the present study, we focused our attention on the cytocompatibility of 5 nm citrate-coated PtNPs with human THP-1 macrophages. THP-1 monocytic leukemia cells can be differentiated into macrophages using phorbol-12-myristate-13-acetate (PMA), as a reliable in vitro model [10] for studying immune cell processes. We compared the morphology and the basal release of inflammatory cytokines and chemokines of macrophages differentiated in the presence of PtNPs with untreated controls. We further analyzed the effects of PtNPs with previously differentiated adherent THP-1 and non-adherent HL60. Our results add important information on PtNPs’ interaction with human macrophage differentiation processes in view of future applications for the ROS scavenging “nanozyme”.

## 2. Results

### 2.1. PMA-Induced Differentiation of THP-1 Monocyte into Macrophages

**Figure 1:**
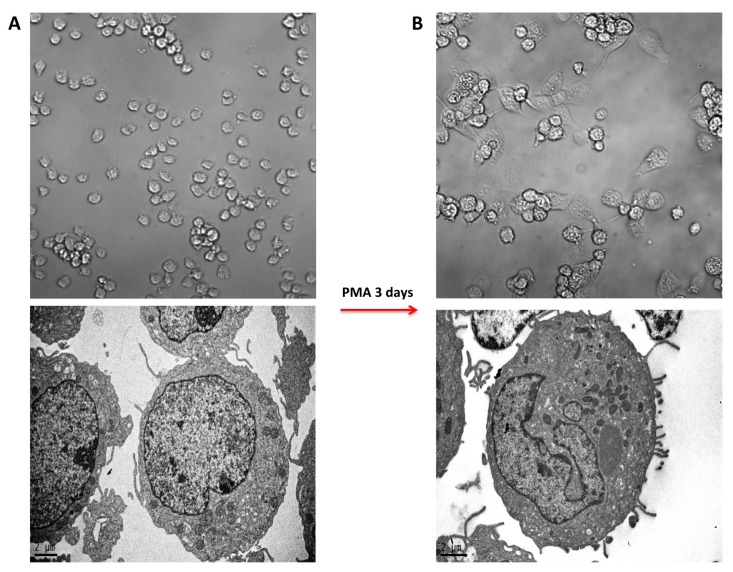
Optical and transmission electron microscopy images of undifferentiated (**A**) and differentiated (**B**) THP-1 cells showing morphological changes induced by PMA treatment.

THP-1 cells were differentiated in vitro into macrophages by the administration of 50 ng/mL PMA for 3 days. The treatment induced the typical hallmarks of macrophages, represented by cell adhesion, spread morphology, increased granularity and irregular nucleus shape, as detected by optical and electron microscopy (Figure 1A,B).

### 2.2. Morphology of PMA-Differentiated THP-1 Cells in the Presence of PtNPs

**Figure 2:**
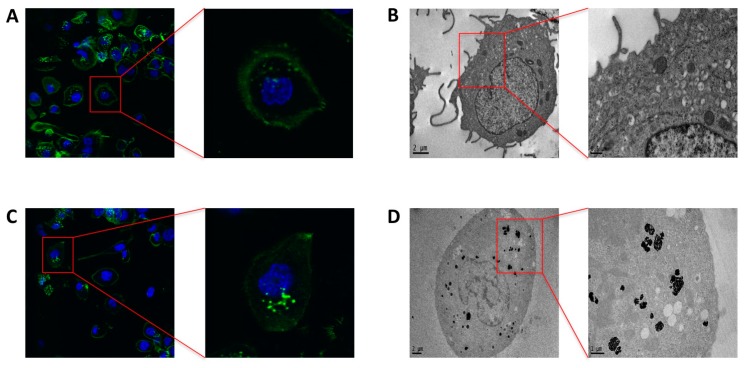
Confocal and transmission electron microscopy images of untreated (**A**,**B**) and PtNP pre-treated (**B**,**D**) differentiated THP-1 cells. Phalloidin staining (in green) shows cytoskeleton organization after PMA induced macrophage differentiation (**A**,**C**). TEM displays intracellular structures in untreated cells (**B**) and PtNP-treated THP-1 (**D**). In Figure 2D, magnification shows highlighted endocytotic vesicles retaining PtNPs.

We assessed the effect of PtNPs on THP-1 differentiation process. The cells were treated with 50 μg/mL 5 nm-PtNPs for 24 h in the monocytic stage and subsequently differentiated by 3-day PMA treatment. Confocal microscopy and Transmission electron microscope (TEM analysis showed no major differences in intracellular structures, cytoskeleton organization or subcellular vesicular compartments between control THP-1 macrophages (Figure 2A,B) or cells pre-treated with PtNPs (Figure 2C,D). TEM images revealed evident NP internalization in monocytic THP-1 treated with PtNPs before PMA-stimulated differentiation (Figure 2D).

### 2.3. Inflammatory Cytokine and Chemokine Release

**Figure 3:**
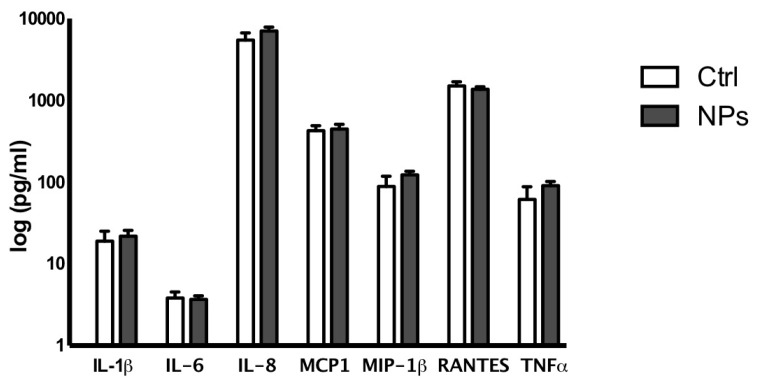
Cytokine and chemokine release by THP-1 differentiated in the absence (Ctrl white bars) or presence (NPs gray bars) of PtNPs. All the data represent the mean ± SD of three independent experiments.

As expected [11], PMA-differentiated THP-1 cells showed increased production of the inflammatory cytokines IL-1β, IL-6, TNF-α and the chemokines MCP-1, MIP1β, RANTES, IL-8 compared to the undifferentiated monocytes (Gatto et al., unpublished data). Interestingly, cytokine and chemokine release by THP-1 cells differentiated in the presence of PtNPs resulted in equivalence to control cells (Figure 3), indicating that the endocytosed PtNPs did not compromise the immune physiology of these cells.

### 2.4. Phagocytosis and Endocytosis of PtNPs in Differentiated THP-1 Macrophages

**Figure 4:**
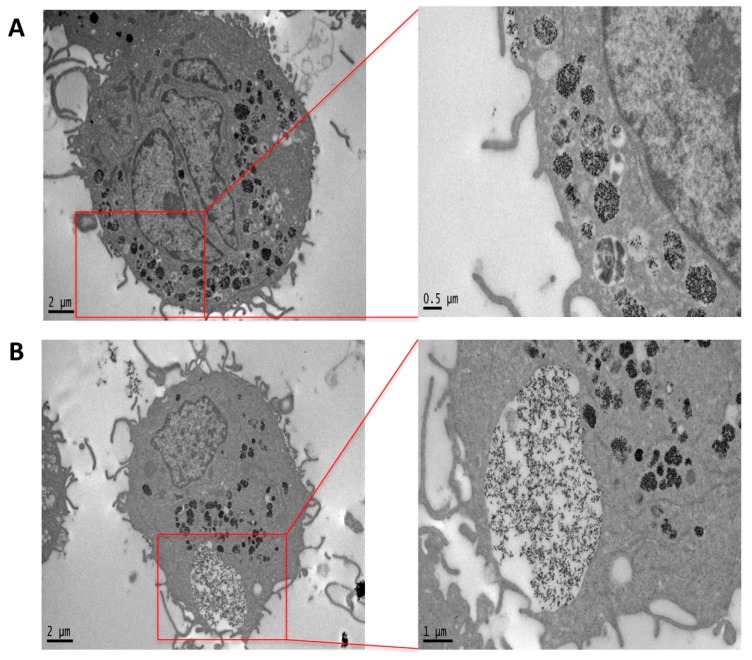
TEM images of pre-differentiated THP-1 cells treated with 50 μg/mL PtNPs for 24 h showing the endocytotic (**A**) and phagocytotic (**B**) process.

TEM images of previously differentiated THP-1 treated with PtNPs for 24 h display huge internalization of NPs. As magnified in Figure 4, two different mechanisms of NP internalization were observed. Figure 4A shows the endocytotic vesicles at different stages of progression. Figure 4B represents the macrophage-specific phagosome formation engulfing several NPs, likely contributing to the enormous internalization of PtNPs observed inside the cells, and mostly confined within intracellular vesicles.

### 2.5. PtNP-Treatment Reduces Metabolism in Previously Differentiated THP-1 Macrophages

**Figure 5:**
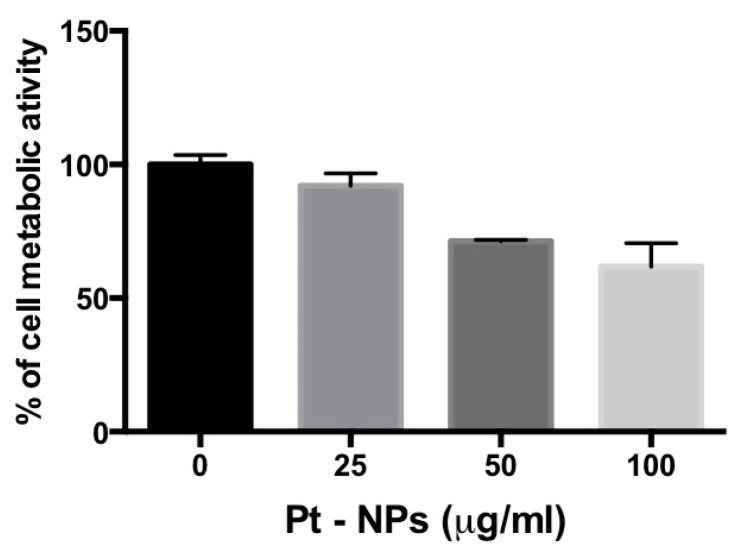
Metabolic activity of pre-differentiated THP-1 treated with different concentrations of PtNPs for 24 h. All the data represent the mean ± SD of three independent experiments.

In order to understand the possible dangerous effect of the observed engulfment of PtNPs on previously differentiated THP-1 macrophages, WST-8 assay was performed 24 h after PtNP exposure. The results showed that increasing doses of NPs leads to a reduction of cell metabolic activity up to 35%, for the highest dose tested (Figure 5). In contrast, the administration of PtNPs in the same concentration range to HeLa cells, which internalize PtNPs only by endocytotic mechanisms, did not provoke similar effects (Figure S1).

### 2.6. Interaction of PtNPs with Undifferentiated and Differentiated HL60

To further understand the effects of 5 nm citrate-coated PtNPs on innate immune cells, we treated a neutrophil-like phenotype with undifferentiated and differentiated HL60 cells. In vitro neutrophil-like granulocyte differentiation was obtained by administration of retinoic acid for 9 days. Fully differentiated non-adherent HL60 showed higher values of side scattering (SSC) and lower values of forward scattering (FSC), as reported in Figure S2. The increase of SSC is in agreement with the higher number of granules in the neutrophil-like phenotype, whereas lower FSC describes the smaller size of the differentiated cells. We considered differentiation of HL60 to be complete when >95% of cells were gating within arbitrarily fixed parameters of SSC and FSC corresponding to granulocyte morphology analysis by TEM.

**Figure 6:**
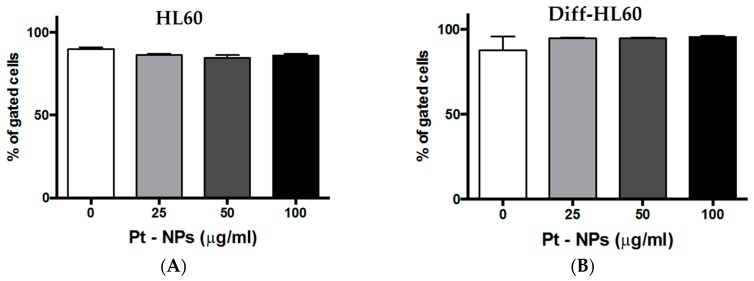

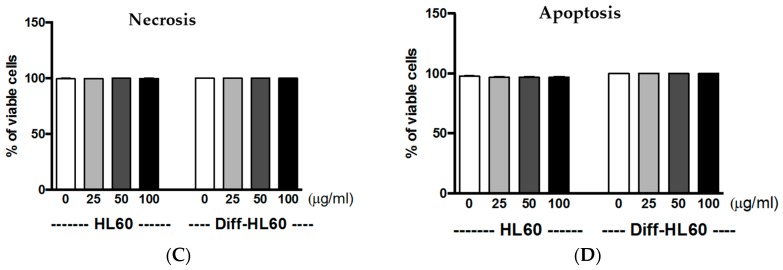
(**A**,**B**) The bar graphs show the percentages of living undifferentiated (**A**) and differentiated (**B**) cells after 6 h incubation with 25, 50 and 100 μg/mL 5 nm-PtNPs. (**C**,**D**) AnnV/PI assay by flow cytometry. (**C**,**D**) Bars show necrosis (**C**) and apoptosis (**D**) of undifferentiated and differentiated HL60 cells, untreated or treated with PtNPs at different concentrations for 6 h. All the data represent the mean ± SD of three independent experiments.

To evaluate the potential cytotoxicity of PtNPs in both non-adherent HL60 phenotypes, an AnnV/PI assay was performed by flow cytometry. We considered the percentage of gated cells in the FSC/SSC living region (Figure 6A,B; Figure S3), then measured the number of necrotic/PI-positive cells and apoptotic/AnnV-positive cells (Figure 6C,D), as reported in the method section. Twenty-five to 100 μg/mL PtNPs were added to the cell cultures for 6 h without inducing any significant necrotic or apoptotic cell death in both the HL60 phenotypes.

### 2.7. PtNP Internalization in Undifferentiated and Differentiated HL60

**Figure 7:**
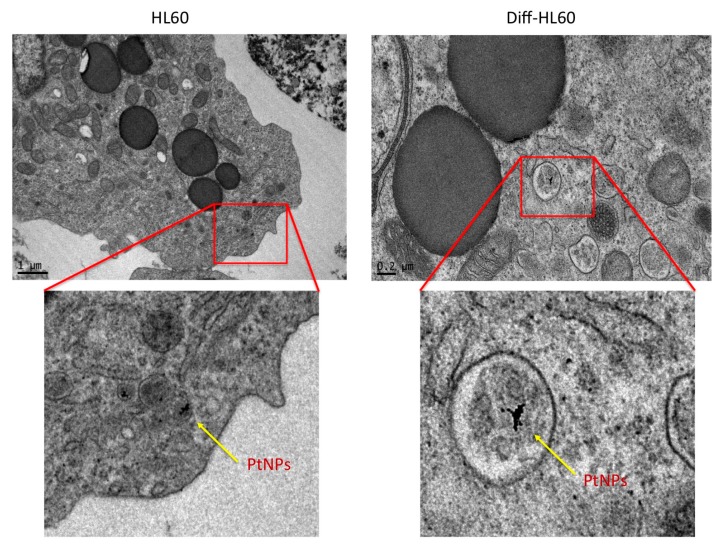
TEM images of undifferentiated and differentiated HL60 treated with 50 μg/mL of PtNPs.

TEM analysis of undifferentiated and differentiated HL60 cells treated with 5 nm citrate-coated PtNPs was performed (Figure 7). Low NP internalization in both non-adherent phenotypes was observed, although stimulation with retinoic acid induced a granulocytic/phagocytic neutrophil-like HL60.

## 3. Discussion

Nanotechnology applications for biomedical research are often limited by the toxicity of several materials [12,13,14]. The development of NPs with intrinsic properties, or as carriers for drug delivery, requires deep investigation of particle-cell interactions, and their potential impairment of cell-cycle processes. Special attention must be given to immune system reactions to NPs, since the absence of immunogenicity will enhance the application in several fields of medicine [15,16].

The innate immune system represents the first active and fast-reacting defense of our body [1]. It is organized firstly to start coordinated enzymatic cascades [17], followed by cellular phagocytosis of the pathogens [18]. The latter is achieved by specialized cells, such as macrophages and neutrophils, that are activated by signals of exogenous and endogenous origin in their surroundings, and subsequently differentiate into precise phenotypes able to build a suitable response for specific threats [19,20,21].

We have already proposed PtNPs as novel catalytic nanomaterials for biomedical applications [8]. Their surface chemistry allows intracellular ROS scavenging and impairment of inflammatory pathways [9], suggesting PtNPs as antioxidant and anti-inflammatory “nanozymes”. Although very promising, PtNP application has been slowed by conflicting results on their toxicity [8,22]. Most of the available data report the use of differently synthesized particles or particles with diverse coatings [7]. The toxicological and immunological impact of PtNP is also influenced by the presence of synthesis by-products or contaminants in the colloidal suspensions, increasing the difficulty of a precise overview [23].

In the present report, we investigated the differentiation of THP-1 macrophages in vitro in the presence 5 nm PtNPs. As a delicate and fundamental process of the immune response, we considered potential alterations to PtNP-treated macrophage intracellular morphology (Figure 2 and Figure 4), cytoskeleton organization (Figure 2), and inflammatory cytokine production (Figure 3). We did not find significant differences in these parameters between treated and untreated THP-1 cells. PtNPs internalized before PMA-induced differentiation did not change either the structure of the cytosolic organelles, or the release of cytokines. Unmodified cytokine release in PtNP-treated cells would seem in conflict with the results obtained by Rehman and colleagues using RAW 264.7 murine macrophages [9]. However, our data refer to inflammatory cytokine and chemokines produced by human THP-1 stimulated for 48 h with PMA, which triggers many pro-inflammatory pathways, leading to the adherent macrophage phenotype and its immunological profile. Murine RAW 264.7 cells are macrophages that do not need PMA for their phenotype in vitro, showing a different and species-specific cytokine release profile.

We also investigated the effects of PtNPs on previously differentiated THP-1 macrophages (Figure 4 and Figure 5). The metabolic decrease observed in these post-mitotic cells (Figure 5) in the presence of PtNPs is likely due to the huge number of particles internalized at concentrations higher than 50 μg/mL by the phagocytosis mechanism (Figure 4), since no toxicity is present during the differentiation of monocytes (Figure 2) or in adherent non-phagocytic HeLa cells tested for the same PtNP concentrations (Figure S1).

To extend the observation of PtNP effects on innate immune cells, we exploited undifferentiated human HL60 cells, and differentiated them into a neutrophil-like phenotype (Figure 6). As reported in Figure 7, 5 nm citrate-coated PtNPs do not show toxicity in both HL60 phenotypes. Interestingly, PtNP internalization in differentiated and undifferentiated cells is similarly low (Figure 7), indicating that it is not increased by the phagocytic phenotype. This may be due to the non-adherent nature of these cell lines, and the shorter time of PtNP exposure. We chose a 6 h treatment for HL60, since the fast proliferation time of undifferentiated cells [24] would have diluted the initial NP concentration-per-cell over 24 h. The possibility of contacts with NPs for HL60 is much lower than for THP-1 macrophages, as differentiated THP-1 firmly adhere to the flask bottom, with a spread morphology (Figure 1) that increases the adhesion of the particles on their surface membrane, thus enhancing phagocytosis. Nevertheless, PtNP-induced toxicity was seen neither for the differentiated THP-1, showing high NP endocytosis (Figure 2), nor for HL60 exhibiting low NP internalization ability (Figure 7).

In summary, our data show that endocytosed citrate-coated PtNPs are highly cytocompatible, and do not impair PMA-induced THP-1 macrophage differentiation processes in vitro. These results add important information in view of nanotechnology applications in biomedical sciences.

## 4. Materials and Methods

### 4.1. Nanoparticles

5 nm Citrate-capped PtNPs, synthesized and characterized as previously reported [4].

### 4.2. Cell Culture

THP-1 (ATCC, Manassas, VA, USA) were grown in RPMI-1640 (Thermo Fisher Scientific, Waltham, MA, USA) supplemented with 10% FBS (Thermo Fisher Scientific, Waltham, MA, USA), 1% Penicillin-Streptomycin (Sigma-Aldrich, Saint Luis, MO, USA) and 0.05 mM 2-Mercaptoethanol (Gibco—Thermo Fisher Scientic, Waltham, MA, USA) in a 5% CO_2_ humidified atmosphere at 37 °C. HL60 cells (ATCC Manassas, VA, USA) were grown in the same conditions, excepting 2-Mercaptoethanol.

### 4.3. Differentiation Assay

THP-1 cells were differentiated with 50 ng/mL phorbol-12-myristate 13-acetate (PMA, Sigma-Aldrich, Saint Luis, MO, USA) for 3 days. After this time, the cells were refeeded with fresh medium without PMA for 2 days to allow cell recovery. Cell differentiation was verified by evaluating cell adhesion and spreading under an optical microscope.

HL60 cells were incubated with 1 μM all-trans retinoic acid (ATRA) (Sigma-Aldrich, Saint Luis, MO, USA) for 9 days to obtain complete differentiation in neutrophil-like cells. Differentiation along the granulocytic pathway was monitored by characteristic changes in morphology using the flow cytometry with MACSQuant Analyzer (Miltenyi Biotec, Bergish, Germany).

### 4.4. Transmission Electron Microscopy

Suspension cells (THP-1, HL60 and differentiated HL60) were incubated with 50 μg/mL PtNPs for the proper time, then washed twice with RPMI and fixed for 45 min in a fixative solution (2% Glutaraldehyde in complete culture medium). The samples were centrifuged and the pellet fixed again with 1.5% Glutaraldehyde solution in Na-Cacodylate buffer 0.1 M. A final post-fixation (2 h) in 1% OsO_4_ solution in Na-Cacodylate buffer 0.1 M was performed. The fixed samples were stained overnight in a 1% Uranyl acetate aqueous solution at 4 °C. Samples were washed in water and completely dehydrated with a scale of Ethanol, transferred in Propylene Oxide and finally infiltrated with epoxy Spurr™ (SPI-Chem, West Chester, PA, USA) resin. Once the resin had hardened for 48 h in oven at 65 °C, thin sections were cut with a Leica EM UC6 ultra-microtome. Adherent cells (differentiated THP-1) were incubated with 50 μg/mL PtNPs for 24 h, washed twice and fixed for 1 h in 1.5% glutaraldehyde in 0.1 M sodium cacodylate buffer. The samples were washed with the same buffer, post fixed in 1% osmium tetroxide in 0.1 M sodium cacodylate buffer and en bloc stained with 1% uranyl acetate aqueous solution. The cells were then dehydrated in a graded series of ethanol and embedded in epoxy Spurr™ resin. Semi-thin sections of the cell monolayer were cut with an ultra-microtome. TEM images were collected with a Jeol JEM 1011 (Jeol, Tokyo, Japan) electron microscope (Electron Microscopy Facility—Fondazione Istituto Italiano di Tecnologia, Genova, Italy), operating at an acceleration voltage of 100 kV, and recorded with an 11 Mp fiber optical charge-coupled device (CCD) camera (Gatan Orius SC-1000).

### 4.5. Confocal Microscopy

THP-1 cells were incubated with 50 μg/mL PtNPs for 24 h and then differentiated with PMA for the proper time. After the recovery, the cell layers were fixed with 4% paraformaldehyde for 20 min at room temperature, permeabilized with 0.1% Triton ×100 in PBS for 5 min and blocked with blocking buffer solution (1% bovine serum albumin in PBS) for 30 min. Then, the cells were incubated in the dark with 0.1 nM Alexa Fluor™ 488 Phalloidin for 30 min for actin microfilaments staining and Hoechst 33342 (Thermo Fisher Scientific, Waltham, MA, USA) 5 μg/mL for 5 min for cell nuclei staining. Confocal microscopy images were acquired by a confocal microscope (Leica TCS-SP5) with an oil-immersion 63× objective, 405 and 488 nm excitation laser wavelengths and a resolution 1024 × 1024 pixels. Z-sectioning images were acquired with a z-slice thickness of about 0.7 μm.

### 4.6. Cytokine Release

THP-1 cells were incubated with 50 μg/mL PtNPs for 24 h, then washed twice and resuspended in PMA for the differentiation. After the recovery time, IL-1β, IL-6, IL-8, MCP-1, MIP1β, RANTES and TNF-α release by differentiated THP-1 cells were quantified using the Bio-Plex MAGPIX Multiplex Reader (Bio-Rad, Hercules, CA, USA) according to the manufacturer’s instructions.

### 4.7. Metabolic Activity

Differentiated THP-1 cells were incubated with 25, 50 and 100 μg/mL PtNPs for 24 h. After the incubation, the cells were washed twice and the cell metabolic activity was evaluated using a standard WST-8 assay (Sigma-Aldrich, Saint Luis, MO, USA).

### 4.8. Annexin-PI Assay

Cell viability was quantified by using Annexin V-PI assay (Miltenyi Biotec, Bergish, Germany) according to the manufacturer’s instructions. In brief, HL60 and differentiated HL60 cells were incubated with 50 μg/mL PtNPs for 6 h. After the treatments, the cells were washed and incubated with Annexin V-FITC for 15 min in the dark at room temperature. Subsequently, the cells were washed and PI solution was added immediately prior to analysis by flow cytometry with MACSQuant Analyzer. The percentage of necrotic or apoptotic cells was evaluated using MACSQuantify software.

### 4.9. Flow Cytometry

For the evaluation of cell viability, HL60 and differentiated HL60 cells were incubated with 50 μg/mL of 5 nm PtNPs. After 6 h incubation, the effect of PtNP internalization on cell viability was evaluated by flow cytometry with MACSQuant Analyzer (Miltenyi Biotec, Bergish, Germany) using MACSQuantify software.

## 5. Conclusions

We demonstrated that 5 nm PtNPs show a good degree of cytocompatibility and do not alter PMA-induced THP-1 differentiation in vitro, as shown by morphological analysis of the cellular structure. Moreover, the release of inflammatory cytokine and chemokines by THP-1 cells differentiated in the presence of PtNPs is not different from the same cells differentiated in the absence of particles. Although the viability of undifferentiated and differentiated neutrophil-like HL60 is also not affected by PtNPs, the particles internalized within these cells seem very few, emphasizing the specificity of PtNP-immune cell type interaction. All these results contribute to the knowledge of PtNP interaction with immune cells in view of their potential applications in nanomedicine.

## Supplementary Materials

The following are available online at www.mdpi.com/2079-4991/7/10/332/s1, Figure S1: WST-8 assay of HeLa cells treated with increasing concentration of PtNPs for 24 h, Figure S2: Flow cytometer dot plots and TEM images of internalized undifferentiated and differentiated HL60, Figure S3: Dot plots show the comparison between untreated and PtNP-treated differentiated HL60 for 24 h.
