# Supplementary material for: PMA-Induced THP-1 Macrophage Differentiation is Not Impaired by Citrate-Coated Platinum Nanoparticles
Source: Nanomaterials (Basel). 2017 Oct 17;7(10):332. doi: 10.3390/nano7100332 (PMC5666497; doi:10.3390/nano7100332)
Supplement: Supplementary file 1 [file nanomaterials-07-00332-s001.pdf]

PMA-Induced THP-1 Macrophage Differentiation is Not Impaired by Citrate-Coated Platinum Nanoparticles

Francesca Gatto [1,2], Roberta Cagliani [1,3], Tiziano Catelani [4], Daniela Guarnieri [1], Mauro Moglianetti [5], Pier Paolo Pompa [1] and Giuseppe Bardi [1,*]

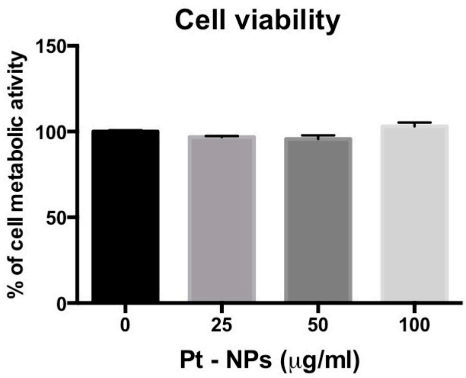

Figure S1. WST-8 assay of HeLa cells treated with increasing concentration of PtNPs for 24h.

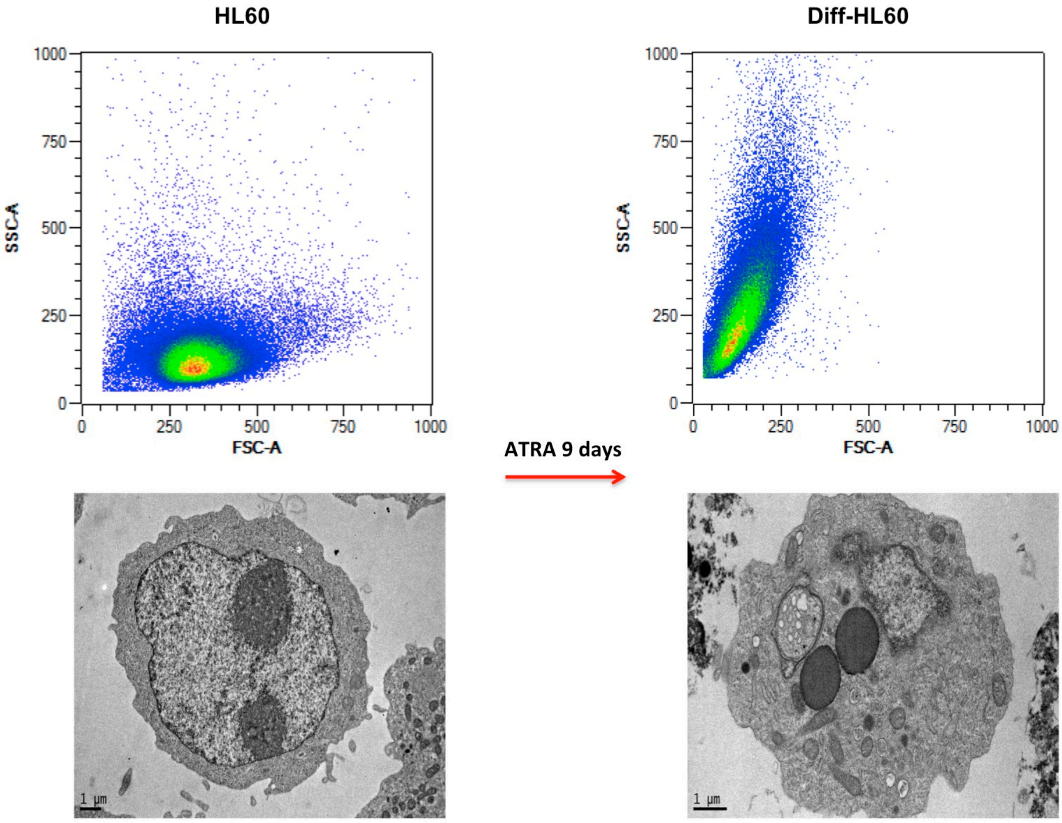

Figure S2. Flow cytometer dot plots and TEM images of internalized undifferentiated and differentiated HL60.

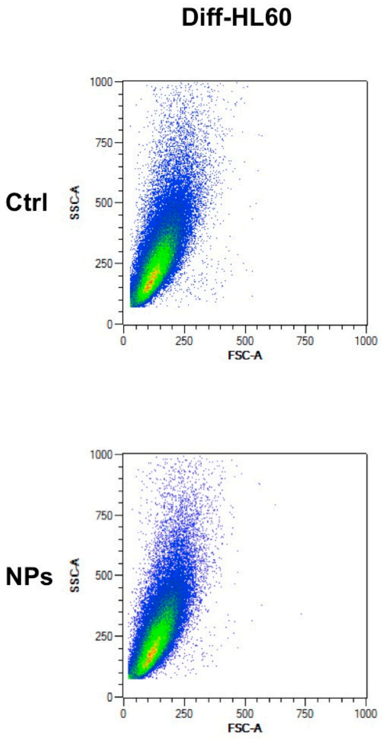

Figure S3. Dot plots show the comparison between untreated and PtNP-treated differentiated HL60 for 24h.
